# Explicit and implicit issues in the developmental cognitive neuroscience of social inequality

**DOI:** 10.3389/fnhum.2012.00254

**Published:** 2012-09-06

**Authors:** Amedeo D'Angiulli, Sebastian J. Lipina, Alice Olesinska

**Affiliations:** ^1^Department of Neuroscience, Carleton UniversityOttawa, ON, Canada; ^2^The Institute of Interdisciplinary Studies, Carleton UniversityOttawa, ON, Canada; ^3^Unidad de Neurobiología Aplicada (UNA, CEMIC-CONICET)Argentina; ^4^Centro de Investigaciones Psicopedagógicas Aplicadas (CIPA-UNSAM)Argentina

**Keywords:** EEG, ERPs, fMRI, neurocognitive processes, neuroimaging, socioeconomic status, theoretical neuroscience

## Abstract

The appearance of developmental cognitive neuroscience (DCN) in the socioeconomic status (SES) research arena is hugely transformative, but challenging. We review challenges rooted in the implicit and explicit assumptions informing this newborn field. We provide balanced theoretical alternatives on how hypothesized psychological processes map onto the brain (e.g., problem of localization) and how experimental phenomena at multiple levels of analysis (e.g., behavior, cognition and the brain) could be related. We therefore examine unclear issues regarding the existing perspectives on poverty and their relationships with low SES, the evidence of low-SES adaptive functioning, historical precedents of the “alternate pathways” (neuroplasticity) interpretation of learning disabilities related to low-SES and the notion of deficit, issues of “normativity” and validity in findings of neurocognitive differences between children from different SES, and finally alternative interpretations of the complex relationship between IQ and SES. Particularly, we examine the extent to which the available laboratory results may be interpreted as showing that cognitive performance in low-SES children reflects cognitive and behavioral deficits as a result of growing up in specific environmental or cultural contexts, and how the experimental findings should be interpreted for the design of different types of interventions—particularly those related to educational practices—or translated to the public—especially the media. Although a cautionary tone permeates many studies, still, a potential *deficit attribution*—i.e., low-SES is associated with cognitive and behavioral developmental deficits—seems almost an inevitable implicit issue with ethical implications. Finally, we sketch the agenda for an ecological DCN, suggesting recommendations to advance the field, specifically, to minimize equivocal divulgation and maximize ethically responsible translation.

## Introduction

In the domain of developmental cognitive neuroscience (DCN), the study of poverty and social gradients is a very young area of research where a core consensus is quickly emerging from basic results. However, as any emerging scientific discipline, the approaches used are influenced by epistemological stances inherited from other disciplines, and potentially implicit beliefs systems. Explicitly or inadvertently, such influences can lead this critically important new area of research to methodological and ethical foundational challenges. For example, some of the issues in need of debate are: conceptual and operational definition criteria of socioeconomic status (SES) and poverty, scarcity of specificity on how children experience different types of deprivation in different settings, and scarcity of critical cross-cultural considerations regarding social exclusion mechanisms. Debate on these and other issues involves the building of consensus on interventions aiming at attenuating the effects of socioeconomic disadvantage on children's development. Without a critical analysis of the emerging issues, scientists may dangerously risk the tendency to simplify the complexity that characterizes both phenomena of development and social inequality. The aim of the present paper is to contribute to a debate on the implicit and explicit conceptual and methodological assumptions underlying the current neurocognitive research on social inequality.

Some of the difficulties of studying SES stems from its inherent spanning over many different areas of expertise, which implies the need to establish interdisciplinary efforts. However, often the integration of information occurs only after the building of substantial databases. Particularly, in the context of the study of SES, that database is growing rapidly and there is an urgency to view the complexity of the problems in question from as many levels as possible. As proposed by several researchers in this field (e.g., Anderson, 1999; Bornstein and Bradley, 2003), the principles of convergent and reciprocal causation help to explain how different levels of analysis (i.e., molecules, cells, systems, individual and social behavior), may contain different patterns of interacting risk and protective factors. For instance, a risk factor present in a context of development—e.g., a rearing environment without enough stimulation for cognitive competences—may not be sufficient to cause a perturbation in the typical course of development, but the interaction between such an environment and maternal depression may indeed have that impact (for reviews and discussions of examples of these instances of cumulative/comorbidity effects, see Bradley and Corwyn, 2002; Hackman et al., 2010).

Another goal of this paper is to review the basis of the *deficit assumption* that is possible to identify in some approaches focusing on SES from a neuroscientific perspective. Many researchers working in this field explicitly mention that results—e.g., impact of poverty on cognitive performance or pattern of neural activation—do not imply any reference to a disorder or deficit. However, the model of explanation underlying some strategies of investigation does not go beyond *similarity*, by virtue of which several difficulties that children show in schools or in laboratory assessments are related to particular neural network patterns of activation that have been found in clinical populations and patients. The latter may raise an implicit prejudice or misconception any time it is applied to exclusively support research efforts aimed at finding differences that reflect negative outcomes in terms of disadvantages of one or more groups as compared to a normative group taken as criterion (Boykin and Allen, 2002, 2004). Conversely, in some other instances, it may seem reasonable that differences in patterns of neural processes between small groups of children may be a sufficient condition to infer a deficit-like condition, such as “developmental delay,” even though there are no manifest differences in behaviors that define the deficit or disorder. Such interpretation is generally grounded on an assumption of underlying neurological immaturity. Yet, there is strong evidence against the plausibility of this stance. Neurological immaturity, particularly neoteny, has been long recognized as trademark of neural plasticity in the human species (De Beer, 1930; Fuentes, 2009) and the association between higher levels of cognitive abilities and delayed trajectories of cortical development in the “late bloomers” confirm that immaturity is not necessarily associated with negative outcomes (Shaw et al., 2006). Approaches alternative to the deficit assumption propose to consider the broad range of plastic cognitive processes, which characterizes human population. From this perspective, differences do not always have to correspond to deficits, as shown in intervention studies (Burger, 2010) and in alternative conceptualizations of conditions such as attention deficit and hyperactivity disorder (ADHD) (e.g., Jensen et al., 1997, 2006) and dyslexia (Geschwind, 1982, 1984).

The present paper is primarily concerned with interpreting lower performance in low-SES children, relative to their high-SES counterparts, as an indication of underlying brain deficit. Similarly, the paper is concerned with assigning low SES as a direct, univariate cause of cognitive deficits. In addition, we examine critically the nature of the still vaguely formulated deficit that generally is (explicitly or implicitly) assigned to individuals with low SES. The latter issue is addressed as a question of normativity, according to which *differences* relative to a criterion deemed as “normal” are interpreted as *deficits*. We mainly focus on the developmental human neuroscience literature which relies on findings of differences between compared groups. Our critical view of the deficit attribution does not underestimate the possibility of real deficit. We present a perspective according to which interventions are the main tool to determine real underlying deficits, since several intervention programs show that many of the lags in low-SES children's performance are neither irreversible nor inevitable.

## State of the art and why it is time for epistemological reflection

Undoubtedly, cognitive scientists and neuroscientists would have a tremendous desire to contribute to understand and “solve the problem” of poverty with the powerful analytic tools that such disciplines can afford. Then, it is natural and genuine to foster the interest in accumulating a synthesis of the available knowledge, or the “state of the art,” and translate this knowledge in action and intervention efforts (Posner, 2009).

Examples of this genuine thrust are the recent reviews by Hackman and Farah (2009), Hackman et al. (2010), Lipina and Colombo (2009) and Raizada and Kishiyama (2010), establishing consensus on the evidence base of the neurocognitive science of social inequality. Based on studies by different researchers during the last decade, these syntheses show how some experiences related to low SES or poverty during childhood are associated with behavioral performance in tasks hypothesized to be related to different underlying neurocognitive systems. Specifically, most studies use behavioral and neuroimaging methods to characterize SES disparities in specific neurocognitive paradigms. These studies are interpreted as suggesting that SES is a predictor of differences in neurocognitive performance, particularly of language and executive functions, and that these differences are found in neural processing even when behavioral performance levels are similar.

These timely reviews raise important issues and highlight the necessity of considering poverty and SES in cognitive neuroscience research as a critical developmental priority towards a scientific agenda that includes the following issues: neural plasticity, sensitive periods, epigenetics, vulnerability and susceptibility, exposure to environmental toxins, nutrition, stress response, impact of different forms of poverty on neurocognitive processing, and influences of childhood poverty on neurocognitive functioning during adult life (Hackman and Farah, 2009; Lipina and Colombo, 2009; Gianaros and Manuck, 2010; Hackman et al., 2010; Raizada and Kishiyama, 2010). That is, finally SES is a legitimate topic for serious neurocognitive scientific investigation rather than being relegated to the role of control or confound variable.

However, as in all areas of recent development it would be now necessary to also begin the review of some important epistemological and ethical issues. For instance, some of the evidence included in the mentioned reviews deserves to be explored applying alternative interpretations, and consequently contribute with the enrichment and complexities of the neuroscientific agenda. In particular, it is unclear the extent to which the available laboratory results may be interpreted as showing that low-SES related experiences may be necessarily associated with cognitive and behavioral disorders or deficits in any environmental and cultural context; and how findings may be interpreted to inform the design of different type of interventions—particularly those related to educational practices.

Although a cautionary tone permeates the mentioned reviews, still, a potential *deficit attribution*—i.e., the notion that poverty or low-SES is associated with cognitive and behavioral developmental deficits—seems almost an inevitable implicit issue (Boykin and Allen, 2002, 2004), particularly when media and scientific divulgators approach the theme even after talking with their authors (e.g., Sanders, 2008).

Despite the preliminary description of cognitive processing in terms of basic operations—which represent a scientific advance with multiple scientific and policy implications—as it could probably said by most current literature in the field, these reviews do not distance enough from a recent relative of psychometric tradition that has systematically found confirmation of intellectual and cognitive delay as part of the typical profile of disadvantaged children (e.g., Jensen, 1968). Like forty years ago, the accumulation of evidence for negative outcomes can be perceived as overwhelming, so much so that it might be recognized almost as the same “syndrome of poverty” representation (Lewis, 1967) systematically resurfaced to the arenas of social, behavioral and psychological disciplines for four decades since. Indeed, the current themes of discussion concerning research and intervention resemble strikingly those historical precedents and context when social disadvantage in children was explicitly linked with *deficits from cultural deprivation* (e.g., Hunt, 1968; see also Deutsch et al., 1968). At this point, then, epistemological, conceptual and methodological reflection is timely in order to better understand the implications of low cognitive performance in today's children everyday life and any type of intervention effort, even those conducted in laboratory contexts, and the groups of children and families that are targeted. In the following sections, we explore the nature of the evidence of SES disadvantages in current DCN, starting from the conceptualization of poverty and SES used in neuroscientific studies.

### Issues about the definition of poverty

Social stratification notions such as “class,” and the different versions of “poverty” are classic constructs for sociology, economy, psychiatry, and several other disciplines that participate in analyzing poverty phenomena. Nevertheless, there is no unique and definitive definition of general poverty, particularly, of childhood poverty (Minujin et al., 2006). It is also important to point out that there is an ongoing debate on the inconsistency with which SES is being measured in studies across the developmental sciences. Some studies have used questionnaires (e.g., Hollingshead's *four factor index of social status*), others inferred SES through the parents' educational level, the number of TV or computers at home, and others inquired about the income of the family directly. Furthermore, some studies relied on a single key proxy, such as a measure of family income, while others have used composite variables or multivariate approaches with inclusion of indicators ranging from few (e.g., five) to a large number (e.g., 25). At present, it is not clear exactly the extent to which the definitions and measures of SES are comparable, if they capture the same underlying factors and whether the results that are obtained with such definitions and measures can be legitimately synthesized under the same unitary interpretation (Duncan and Magnuson, 2012). In this section, we will selectively review just some of the inconsistencies as they apply specifically to DCN studies (for more detailed discussions see Bradley and Corwyn, 2002; Minujin et al., 2006; Bornstein and Bradley, 2003). For our scope, we will use *low SES* as synonymous of “relative poverty,” however, we will use *poverty* in the instances where we want to refer specifically to “absolute poverty.”

One of the most common approaches uses the specific level of income of a family to define a child's individual SES. There are inherent problems with this approach, because it views the effects of SES from a monetary standpoint and ignores the cofactors, which sometimes are better predictors of the graded effect of low SES. For example, prenatal factors such as the maternal mental health have an enormous impact on the cognitive development of a child, and cannot be reflected in the annual income of a family (Surkan et al., 2011). This approach also does not consider the composition of households in terms of gender and ages, which could adversely affect the consideration of women and children needs. For instance, it does not take into account that children have much different needs than an adult, and that the goods of the household might not be split evenly among its members (Minujin et al., 2006). More importantly, many of the things that are crucial for children healthy development are unrelated to purchasing power, and are not based on income criteria. A poverty-stricken family might still have access to clean air and water or other freely available resources. However, most poor families in many countries live in areas with toxins in their proximal environment, which have been proven to have a deep-rooted impact on human well-being since prenatal stages (Hubbs-Tait et al., 2005). For example, prolonged exposure to nitrogen dioxide—a common traffic air pollution- has been associated with reduced performance in tasks demanding working memory, motor function and coordination (Freire et al., 2010). Furthermore, severe exposure to air pollutants in urban environments (i.e., fine particulate matter <2.5 μm in diameter, PM_2.5_) has been linked with children's cognitive impairments (Calderón-Garcidueñas et al., 2011a), white matter neuropathology (Calderón-Garcidueñas et al., 2012a,b), brain stem and auditory processing pathology (Calderón-Garcidueñas et al., 2011b) and neuroinflammation (Calderón-Garcidueñas et al., 2012a,b).

This widely used definition of poverty is being challenged by other multifactorial approaches such as the one informed by the universal principles of human rights (Barbarin, 2003; Roosa et al., 2005; Minujin et al., 2006). Human rights-based approaches usually include quantitative and qualitative indicators of access to education, health and work market, among others, and could offer a more consistent way to define poverty (UNPD, 2010). The income approach is an indirect measure of SES because it uses parental income and household wealth to measure the individual status of each child. The human rights-based approach can be applied to each child and family separately, and is a more accurate portrayal of their conditions, by being more sensitive to individual factors. This approach has also the potential to contribute to the discussions regarding the deficit assumptions in the context of the scientific community (see above), because it proposes to think about deprivations in terms of a continuum with several possible outcomes. Furthermore, in an ethical context of discussion considering poverty in terms of human rights requires to review critically the constructs of deficits, disability and potentialities.

However, even such an approach must be modified to accommodate for the different needs of children at different stages in cognitive development (Lipina et al., 2011). There is a gradient of effect according to several crucial factors such as: which risks the child was exposed to, the length of the exposure, and the child's developmental period (Evans, 2003; Gassman-Pines and Yoshikawa, 2006; Hall et al., 2010). Consequently, the definition of poverty should reflect graded effects of social inequality in interaction with developmental time-course of the child, instead of following the Extreme Group Approach (EGA; Preacher et al., 2005) in which the SES gradient is cut off generally on two halves fixed in time: poor or low-SES groups versus middle-high SES groups. It is known that EGA reduces reliability by exaggerating the differences between the groups and the individuals thereby represented.

### Issues about validity, deficit attribution, and assignation of group normativity

As we have already mentioned, for decades, cognitive differences between poor and non-poor or low and high SES developmental samples have been reported in psychometric and educational tests, such as those measuring IQ and Developmental Quotients (e.g., Bayley, CAT-CLAMS), arithmetic and language performances (Duncan and Brooks-Gunn, 1997; McLoyd, 1998; Bradley and Corwyn, 2002). However, historically, the interpretation of IQ differences and what they really mean is still debated (Ceci, 1996; Nisbett, 2009; Stanovich, 2009). The main line of criticism is that, if interpreted as a pure measure of intelligence, as in the classic psychometric tradition, IQ tests may be biased against certain groups, which predominantly include children at the lower end of the SES spectrum.

In his review of the WISC-R—one of the most widely used IQ tests in children—Sattler (1992), proposed that most of the evidence shows that mean WISC-R scores differences between groups often confound SES and cultural backgrounds. That is, minority children may not be culturally prepared to take IQ tests, because they may not appreciate the demands, achievement stimuli, time pressures, competitive edge required, and may not see the test in the same way. Furthermore, he observed that although most IQ tests correlate with performance on educational achievement tests and, therefore, as concluded by Neisser et al. (1996; p. 93) may be said to have no “predictive bias,” achievement tests are known to be unfair to certain groups in which low SES and minority status is overrepresented. Thus, the correlation between educational achievement and IQ scores could be interpreted not in terms of predictive validity but rather as a confirmation of another sense of test bias, by some called “outcome bias” (Neisser et al., 1996; p. 93) by others related to “cultural bias” in the construction and administration of the tests (Suzuki and Valencia, 1997; Suzuki and Aronson, 2005) and yet by others differentiated as “fairness” (Helms, 2006).

In addition, a different but related line of behavioral research, has demonstrated that minority groups are especially vulnerable to the effects of social status *stereotype threats* during IQ testing (Steele and Aronson, 1995; Steele, 1997). Very recently, Kishida et al. (2012) confirmed that the modulation of IQ performance resulting from framing the test-taker's environment with explicit or implicit cues about the test-taker's stereotyped social status is correlated with changes of the BOLD (blood oxygenation level-dependent) responses in amygdala and dorsolateral prefrontal cortex.

If IQ testing reflects the modalities, tasks or abilities contingent with the assessment situation, it follows that the differences between low- and high-SES children on some IQ subtests do not necessarily or predominantly reflect differences in the assumed complex neurocognitive operations. Other types of neurocognitive processing could be involved when “background” motivational, social or noisy circumstances appear during an assessment. Thus, although not all the performance differences are biased in the same way (especially those that control for some of these confounders), it is not known the extent to which the underlying cognitive processes are more important than motivational, emotional or social ones during the assessment situation to perform well on IQ tests.

Another criticism is that low SES children may have lower IQ scores because the tests do not adequately capture how those children really function in everyday settings. Elkind (1973) suggested two possible accounts as alternatives to the attribution of deficit. One account may be that children from low-SES may develop more quickly than their high-SES counterpart to anchor all their experiences around problems or tasks that involve practical reasoning, albeit very complex (*premature structuring*). The other account may be that low-SES children may get to the same point as the high-SES children get, in their mental growth, but they may get there using *different* functional pathways (*alternate elaboration*). Hence, interpretation of the effects of low-SES or poverty as a form of intellectual deprivation or lack of cognitive stimulation should be qualified more precisely. Elkind suggested that intellectual deprivation in poor children could refer to the kind and not to quantity of stimulation. What poor children may be deprived of is the same kind of stimulation expected in middle- or high-SES children, the one that will prepare them to do well in tests and school achievements (Elkind, 1973; p. 73). On the constraint of a majority or mainstream group, our culture does value some skills over others, and the IQ tests can be considered an accurate predictor of a person's ability to succeed in the abilities most valued by such a culture (favoring middle- and high-SES, Nisbett, 2009). Because IQ tests are at least in part culturally biased, being a reflection of the dominant culture's values, the most appropriate interpretation seems that likely they are measures of normativity in our population (Vonèche, 2006).

The indeterminacies just outlined put into question that IQ differences can be used as reliable indicators of SES neurocognitive differences, if the normativity issues are not considered in the interpretation of the results.

### Issues about deficit attribution and learning skills: an opportunity for DCN

Careful manipulation of classroom setting and teaching strategies have shown that low-SES children might not be lacking the IQ to perform accurately: approaches to learning are teachable skills that may serve to lessen the gap between advantaged and disadvantaged children in the classroom. These skills are malleable, and therefore susceptible to interventions, and generalized to other areas of children's life (Domínguez et al., 2011). When controlling for stable traits such as intelligence, it has been found that learning processes account for most of the variability of academic success in the classroom (Schaefer and McDermott, 1999). This suggests that in some circumstances, one of the main difficulties of low-SES children could be the fit between their approach to learning and the typical classroom setting.

For instance, to bring the above argument closer to the neuroscience realm, some older conditioning studies (e.g., Bresnahan et al., 1969) have confirmed that low-SES children do not tend to employ the “win-stay, lose-shift” method of learning -the strategy of keeping a hypothesis if it is proven right, and discarding it for a new one if it is proven wrong. Low-SES children tend to preserve a hypothesis based on even partially reinforced behavior, and do not adjust their behavior when proven “wrong”. This failure to adapt to the “win-stay, lose-shift” strategy could be the result of overlearning. The number of confirmations after the child has learned the hypothesis makes it more difficult to shift hypothesis after partial reinforcement is introduced (Bresnahan and Shapiro, 1972). Alternatively, the reason because low-SES children tend to preserve their hypothesis may be due to their inconsistent reinforcement histories, and even if low-SES children's hypothesis is not reinforced 100% of the time, it may be reinforced more than what they are used to (Bresnahan and Shapiro, 1972). In their pivotal study, Bresnahan and Blum (1971) exposed high- and low-SES children to 0, 6, or 12 random reinforcements prior to the beginning of hypothesis formation. At 6 random reinforcements, high-SES children were making almost as many errors as low-SES children. At reinforcement number 12, the high-SES sample performed just as badly as the low-SES sample. This suggests the devastating effect of chaotic reinforcement on children ability to shift and form hypothesis, and may offer a possible explanation regarding why low-SES children have difficulties in this area. Overlearning may also contribute to the gradient of SES effect on cognitive development, since the longer a low-SES child spends in a chaotic environment, the harder it is to adapt the structured academic world (Maxwell, 2010). Both groups of children employ cognitive strategies, but low-SES children would be more predictable. They seem to base their responses on the previous reinforcement, rather than attempting to figure out the odds (Silverman and Shapiro, 1970). However, it has been shown that when the more complex shifting reinforcement strategies are rewarded, then all subjects—whether low- or high-SES—switch to them (Bresnahan and Shapiro, 1972), and, in particular low-SES children show a much faster extinction period than their high-SES counterpart when rewards are eliminated.

This line of evidence is consistent with current research approaches on cognitive flexibility (Lipina et al., 2005; Clearfield and Niman, 2012) environmental chaos (Evans and Wachs, 2010) and adaptive executive attention (Mezzacappa, 2004; D'Angiulli et al., 2008a,b). Recently, literacy and numeracy skills have begun to be approached by DCN and Educational Neuroscience as well (Blair and Razza, 2007; Battro et al., 2008; Lipina and Sigman, 2012), constituting a fertile field to reanalyze some of the mentioned hypothesis from a neuroscientific perspective. There are now better opportunities to test the continuity between basic skills or preferences of information processing, which sculpt learning very early outside the schools (Blair, 2002), and the ways in which rules are understood and manipulated later on, inside and outside the schools, contributing to how children learn and think in everyday life.

### Issues about deficit attribution, cross-cultural neuropsychology, and the integration of cognitive and brain activation levels of analysis

A branch of neuropsychological research extending the classic work of Luria (1976) has focused on establishing more direct links between families SES as it is embedded in the sociocultural context, especially in literate versus illiterate groups, and neurocognitive functions. Using batteries of neuropsychological tests, Ardila and colleagues (Roselli and Ardila, 2003; Ardila, 2005) have built a knowledge base on the interactions between social environment and the development of neurocognitive abilities during the life-span (for a comprehensive overview see Uzzell et al., 2007).

Nevertheless, behavioral measures do not fare any better since they are indirect and too global to be reliably put in correspondence with circumscribed focal brain deficits of the size picked up by, for instance, magnetic resonance imaging (MRI). The link between a focal lesion and the large dynamic networks could possibly be empirically incommensurable (Logothetis, 2008) and could only be proven by direct manipulation of the “black box” (Farah, 1994), such as systematically and selectively impairing a neural network to observe how the resulting focal brain damage directly affects the intervening process. Thus, these correspondences are at best suggestive and grossly approximate.

Confronted with the issues of indirect, inferred evidence, several investigators have turned to different neuroimaging techniques. One of these, the evoked-related potentials (ERPs), a non-invasive, child-friendly and relatively inexpensive technique, allows researchers to capitalize on the greater sensitivity of ERP compared to behavioral measures, and exploring differences between groups across different measurements of a same construct (i.e., attention). For example, Stevens and colleagues (2009) found amplitude differences between high- and low-SES children to probes in an unattended channel, yet both groups had equivalent comprehension and memory performance for story presented in the attended auditory channel. Thus, these findings may suggest that group differences in distractor suppression arise from differences in attentional modulation at early stages of perceptual processing (i.e., within 100 ms of stimulus presentation), confirming how ERP measures are able to detect differences (i.e., with millisecond accuracy) that may not be observable with the usual behavioral measures used in most laboratories.

In another study by Kishyiama et al. (2009), high- and low-SES groups were equated on standardized norms from neuropsychological tests. The predictions and interpretations of these findings were based on considerable evidence concerning two specific attention-related processes (i.e., novelty and prefrontal-dependent extrastriate responses, e.g., Barcelo et al., 2000; Yago et al., 2004). Consistent with ERP measures of target detection, they found no group differences in behavioral target (novelty) responses (which does not imply that behavioral differences do not exist, as other studies have been verifying in the last decades) but supported the hypothesis that group differences would only be observed in prefrontal-related neural responses. The latter results have been also interpreted as demonstrating that observed differences in prefrontal-related ERP responses cannot be attributed to task difficulty (see Hackman and Farah, 2009).

The most important contribution of these studies is the demonstration of the importance of analyzing the influences of SES on neurocognitive performance simultaneously according to at least two level of analysis: (1) behavior, and (2) concomitant neural activation. However, at the same time these types of analyzes illustrate some epistemological difficulties on what DCN should incorporate in its agenda. Both studies seem to make different assumptions on the measured ERP waves that induce a sort of confusion of the level of analysis of the explananda. It is possible that they confound the level of brain activation with the level of functional organization of complex cognitive or mental events (e.g., cognitive control). The latter cannot be operationalized in well defined sets of single operations, or put in correspondence with large regional changes of activation, which in turn are characterized by large individual differences and are directionally ubiquitous—can correlate with both positive or negative polarities in ERPs (or activation and deactivation in fMRI and PET). There is now a considerable literature demonstrating that no currently available single neuroimaging technique affords such a fine resolution grain to directly portray *how* the flow of information is organized for complex cognition. That is, showing where and the extent the information is used does not elucidate how the brain uses it for cognitive operations (Roland, 1993; Sartori and Umilta, 2000; Servos, 2000; Logothetis, 2001, 2008; Faux, 2002; Krekelberg et al., 2006).

In some neuroscientific reports, it seems that the role of the analysis of behavior and functional organization may be considered as ubiquitous, whether low-SES children do or do not perform similarly to middle or high class children, their brain is almost expected not to be activated as efficiently as it should (see D'Angiulli and Lipina, 2010; and Jensen, 2009 for examples of such an assumption). But in the absence of morphological abnormalities, departure from typical brain activity in low-SES individuals can only be validated in relation to an external objective criterion of low performance. It would be interesting to analyze to what extent these assumptions were or not inherited from the behavioral studies on the effects of SES on behavior, school achievement and IQ, which also have tended to hinge on the effect of normativity. That is, low-SES samples differ from typically developing children belonging to middle-class majority group, therefore, this could be interpreted as a delay, an impairment or just atypical. Another potential implication of this suggested inheritance could be that in some neuroimaging studies is considered as a plus-value point when children from disparate SES backgrounds achieve the same levels of behavioral performance. Despite the advantage of identifying apparently pure neural measures indicating activation differences, such findings do not necessarily imply that neural differences are deficits. In other words, deficits cannot be presumed in samples of low-SES individuals only based on brain differences (Elkind, 1973).

Another type of epistemological issue would be represented by those studies that equate patterns of activation from different types of populations, such as brain-injuried patients, with low-SES children. For instance, in the Kishyiama et al.'s work (2009), the background research (Barcelo et al., 2000; Yago et al., 2004) supporting the brain wave and top-down mechanism considered is based on very controlled but rather small studies with stroke patients and adults. The comparison between stroke patients and children from low-SES backgrounds would present some epistemological issues. First, it did not address how age modulates the associations between SES, behavioral performance, and brain activation. Second, the use of lesion models or references would induce the assumption that dissociation techniques prove localization. However, behavioral changes from damage to a certain part of the brain, does not indicate sufficiency. That is, consistent with *pluripotentiality* (Luria, 1964, 1966) and *neural reuse* principles (Anderson, 2010), the affected areas of the brain could be contributing crucial information, but the changed function may not necessarily be all or even partially represented in that area, but in a chain of complex temporal and spatial networks dynamics. Another example of this argument would be the Noble and colleagues finding that the Left Perisylvian/Language System is shown to have a significant correlation to SES variation (Noble et al., 2006), if by over-interpreting we could forget that the “Left Perisylvian/Language System” was proposed by the authors as a broad operational construct, that correlation could be also put in direct correspondence with differences in focal brain functions in left hemisphere structures specialized for language, such as Broca's area (Raizada et al., 2008). However, although speech functions are assumed to correspond to a decidedly contained region, a large variability in lesion patterns and speech disturbances have been observed (Hojo et al., 1984) and Broca's area has been implicated in functions other than speech, such as tool use (Higuchi et al., 2009). Furthermore, the developmental validity of hypothesized neurocognitive systems such as the Left Perisylvian/Language System really comes from few non-longitudinal studies with results from variable samples, implying that behavioral evidence of neurocognitive differences between SES groups, especially in preschool children, are still tentative at this point.

There is also the issue of a certain circular logic in defining cognitive processes. The research would induce these processes from task-dependent behavior, but the formation of these tasks require prior knowledge of the sought after process. This previous inferred knowledge can taint the experiment and produce results that seem valid, only because they could validate the researcher's expectations in the first place. Such circularity can be particularly problematic for research on SES. For example, in the Stevens and colleagues study (2009), the described cognitive performance is related to a very complex semantic comprehension listening task, which engages various components of working memory, yet the positive wave differential effects which much of their analysis focuses on is interpreted as mostly reflecting early attentional processes. Consequently, it would be important to be cautious before confirming the involvement of only one type of cognitive process, in order to contribute with a reliable epistemological validation of psychological constructs—especially when SES issues are involved for the implications of equally valid alternative conclusions. Finally, some methodological decisions could highlight the negative aspects of different cognitive processes when applying rigorous exclusion criteria that results in samples with disparate health or performance conditions (Hackman et al., 2010).

### Intervention efforts and deficit attribution biases

Although middle- and high-SES children also could experience socioemotional and behavioral/cognitive issues related to atypical development (e.g., Luthar, 2003, 2006; Luthar and Latendresse, 2005; Ansary and Luthar, 2009) they are not frequently and promptly seen as eligible for interventions, if not within the realm of universal public health and education. However, low-SES children are mostly seen as eligible for many types of interventions worldwide. By far this protective attitude is absolutely necessary and warranted, because the social inequities produced by each society systematically violate children basic rights, gradually eroding their developmental opportunities, to the point of being stigmatized and excluded from society (UNICEF, 2005). However scientists need to take responsible decisions regarding the eligibility to intervention, by considering that interventions, especially in the area of DCN, involve several issues that require the inclusion of children from all socioeconomic backgrounds (Jolles and Crone, 2012).

Lack of awareness of the potential biases associated with the deficit attribution and normativity conformism can support practices that perpetuate inequalities and potential neglects. Experimental interventions targeting neurocognitive functions could be speculative if simply arise from proposals based on unfocused evidence of correspondence with brain functions. Rather, it would be important to identify potential contributions of experimental interventions aimed at optimizing cognitive, emotional and learning processes of low-SES populations to foster inclusion in their community and institutions.

At present, the available evidence about the cognitive gains after training in laboratory contexts [e.g., Rueda et al., 2005 (attentional training/healthy children); Wilson et al., 2006, 2009 (arithmetic training/dyscalculic children); McCandliss et al., 2003; Temple et al., 2003; Shaywitz et al., 2004 (attention and phonological awareness/dyslexic children)], or school/community intervention programs aimed at optimizing cognitive development (e.g., Perry Preschool, Abecederian, Chicago CLS, Tools of the Mind, Harlem Children's Zone, etc), suggest that neurocognitive plasticity, even considering the current limitations in knowledge and specificity, could be modulated through multimodular complex interventions which include sociocultural contextual variables. Therefore, any statement that implies the idea of a low performance-physiology associated with a lesion process is not necessarily correct. Both can be circumstantial and more studies and new methodologies are needed to better understand several related issues. In this sense, DCN would benefit from multimodular programs that have been shown to be effective in improving low-SES children social inclusion, such as Tools of the Mind (Bodrova and Leong, 2001), Harlem Children Zone (Raver et al., 2008; Tough, 2008).

### Media implications of deficit attribution and normative biases

Media divulgation of DCN studies on childhood poverty could also disseminate the deficit attribution and normativity assumptions to the public. The latter, in turn, can induce the generation of *myths*—as commonly held, but erroneous beliefs—about brain development and the influences of environment and parenting. Once consolidated, the myths require much effort to be eradicated, often requiring the involvement of many professionals, including the neuroscientists (Bruer, 1997). Among the reasons that facilitate this type of undesirable effects, is the lack of discussions among scientists on how to inform media about research findings, although some important efforts have been made in the last years (see Thompson and Nelson, 2001; Illes et al., 2010).

An example of such a dangerous dissemination is an article by Sanders (2008) appeared under several online press subsidiary outlets with associated links and citations which currently still give thousands of hits in Google. While reporting about the study by Kishyiama et al. (2009), the journalist first mentions the following comment by one of the authors: “Kids from lower socioeconomic levels show brain physiology patterns similar to someone who actually had damage in the frontal lobe as an adult.” The quote continues: “We found that kids are more likely to have a low response if they have low SES, though not everyone who is poor has low frontal lobe response.” Then, another author is reported to have said, “These kids have no neural damage, no prenatal exposure to drugs and alcohol, no neurobiological damage… ” “Yet, the prefrontal cortex is not functioning as efficiently as it should be. This difference may manifest itself in problem solving and school performance…” “Those from low socioeconomic environments showed a lower response to the unexpected novel stimuli in the prefrontal cortex that was similar … to the response of people who have had a portion of their frontal lobe destroyed by a stroke.”

To the extent that the alleged verbatim quotes could be trusted, the reference to an association between deficit (prefrontal impairment) and poverty seems clear. However, the results of one single laboratory experiment—with small sample of children from different SES and confounding mixed minority backgrounds—cannot support definitive conclusions. It seems that the mentioned potential mistake in the distinction between different levels of analysis (i.e., behavior, neural activation)—which deserves to be an issue of discussion among scientists in the realm of DCN—may have been omitted from the discussion. Thus, this lack of clarity in the distinction potentially induces to misleading knowledge building and/or scientific divulgation about what poverty really means in the life of children that suffer it and the reversibility or not of the behavioral and neural activation findings.

The same could be said regarding the scientific foundations of interventions based on brain studies. For example, in the same interview, another author is reported having said: “But changing developmental outcomes might involve something as accessible as helping parents to understand that it is important that kids sit down to dinner with their parents, and that over the course of that dinner it would be good for there to be a conversation and people saying things to each other.” This statement includes many assumptions that should be discussed critically in the neuroscientific agenda. First, those kinds of intervention statements probably are true but could fit quite loosely the findings of the many intervention studies that have been conducted in the last five decades, rather than specifically in the emergent field of childhood poverty and brain development. Second, as mentioned, there are many environmental and cultural constraints that could put in question the plausibility of what is suggested parents should do or be like, since poor parents in many developing countries cannot chose to freely engage in those actions (UNICEF, 2012).

To complete the circle, developmental cognitive neuroscientists should also consider that they may not just be at the input stage of the vicious cycle. They may be, like journalists and everyone else in this media-dominated society, also at the receiving end. It is striking that many of the implicit assumptions discussed in this paper could be predicted by watching popular TV shows, such as The Simpsons. Low-SES working class individuals are often portrayed as unintelligent, hence having poor taste, as well as lazy, and incompetent—especially as parents and providers—and cognitively rigid, in political and religious senses (Alper and Leistyna, 2005; Leistyna, 2009). Also, it would be hard to miss the analogy with intervention in the many reality shows that focus on improving the low-SES individuals through “makeovers” targeting all the areas that portray the TV stereotypical social class profiles (Leistyna, 2000; Miewald, 2001; Fink and Lomax, 2012). It is only fair to ask whether any subtle influence creeps into our science feeding back a predisposition to certain default assumptions in the way we approach the very object of investigation. One thing is clear, if media as powerful as TV and online newspapers may already have a background predisposition to consider poverty and low-SES in a certain way, then neuroscientists should be most careful with how to communicate and disseminate the findings, interpretations and conclusions of their studies.

Excellent journalistic reporting of science does exist and is becoming more common even in fields so politically and socially charged as those dealing with poverty (see for example McIlroy, 2010). For effectively translating neuroscience to communities, excellent journalism may very well turn a dangerous weapon into a golden opportunity.

## Deficit attribution, developmental disability and interventions

### From deficit attribution to adaptation

As mentioned, there is no question that low-SES children could benefit from intervention programs aimed at optimizing their developmental opportunities. However, many issues in the realm of interventions should still been analyzed very carefully. For example, it seems that the immediate gains of some interventions go through a “fade out” process. That is, while the results show clear gains within the first months after ending interventions (higher IQs, particularly), successively, these benefits seem to fade and disappear when the children are retested years later (Raizada and Kishiyama, 2010). Interestingly, although IQ points seem to revert back to baseline, children that attended intervention groups seem to have acquired a sort of “grit”: a larger percentage of them later in life are employed, graduate from secondary schools, end up in stable and good employment, and have enough purchasing power to afford dwellings and other personal properties (Knudsen et al., 2006).

The pattern of “gains-losses-gains” has many different kinds of implications. Regarding the neuroscientific agenda in the study of childhood poverty, there is a need to design studies that will permit to explore and analyze what kind of plasticity patterns could explain such changes.

This area of research also implies the analysis of plastic processes related to potential sensitive periods for many aspects of emotional, cognitive, language and social development. From another perspective, this pattern of gains and fading processes also justifies the need to analyze what kind of intervention contents in each module of a program is related with what type of outcome. For example, are the intervention programs that obtain these long-term desirable outcomes more oriented to self-discipline or commitment to learning, or social values? In such a case, any DCN agenda focusing on SES not only should include genuine interactions with disciplines which feature preeminently in the now so-called *learning sciences* (such as education, sociology, and anthropology) but also disciplines that offer alternative frameworks of explanation (for example, population genetics, ecology, and evolutionary biology). Furthermore, as Jolles and Crone (2012) have highlighted, many confounding factors should be considered when interpreting training effects, mostly falling in two categories: (1) *general confound factors*: familiarity, expectancy effects, shared components between the context of trained and transfer tasks, motivation, feedback and reward, and cohort effects; (2) *factors specific to neuroimaging*: such as task performance, task irrelevant processing, awareness of task, morphological changes, “scanner” anxiety, and performance on the scanner.

Many current intervention programs (see section “Intervention efforts and deficit attribution biases”) conceived within DCN have been designed to target preferential neurocognitive functions involved in learning acquisition and basic cognitive operations deemed to support literacy, numeracy and social skills. These interventions were at first conceived for the entire population of children, but most recently there has been a shift to tailor interventions for low-SES children with the rationale that their basic skill development could be optimized (Lipina and Colombo, 2009). The priority has become preventing developmental learning/achievement disabilities that have been found to covary with or be outcomes of SES (e.g., Bradley and Corwyn, 2002). The relationships that connect intervention and SES via the concept of disabilities are instrumental to have a concrete term of reference in place of the vague, fuzzy “deficit-like” condition that looms behind the deficit attributions. Hypothetically, we can assume that the deficit underlying underperformance and non-normative brain responses in low-SES children may, in a form or another, end up manifesting as a developmental disability. Then, we could begin to address the question of how deficit would be concretely defined, understood and dealt with from a perspective that integrates DCN and allied disciplines.

Indeed, the model of deficit that underlies ongoing debate on the construct and status of developmental learning disability in some disciplinary arenas, i.e., education, has shifted the focus increasingly to what resembles an “adaptionist” conception of human abilities and performance (Levine, 1992; Gardner, 1999; West, 1999; Sternberg, 2000; Kalbfleisch, 2004). According to this focus, every *neurocognitive preference* has strengths and weaknesses, depending on the conditions at hand (Blair, 2002; Schibli and D'Angiulli, 2011), which fits the model of parallel non-mutually exclusive continua that could go from dis-ability to hyper-ability (giftedness) depending on the structured interactions between specific experiences and environments in which individuals grow, live and go to school (Maggi et al., 2004; D'Angiulli et al., 2004a,b)—what decades ago Barker (1968) grouped under the term “behavioral settings.” Nevertheless, the spectrum of potentialities apparently is preserved in order to effectively supply our population with a diverse repertoire of adaptive possibilities (Geschwind, 1982, 1984; Gilger and Hynd, 2008). As examples, let us consider two of the most typical developmental disabilities that are increasingly consistent with the adaptionist scenario (West, 1999): ADHD and dyslexia.

ADHD is characterized by inattentiveness, hyperactivity and impulsivity (American Psychiatric Association, 2000). Like in the considerations of low-SES, impairment of executive function is the most prominent account for ADHD symptoms. This hypothesis, however, is criticized because, despite the substantial database of behavioral and neurological tests on ADHD, there are many contradicting findings (Willcutt et al., 2005). For example, memory is one of the supposed deficits. However, this seems to be context-dependent for working memory (Lawrence et al., 2002) or non-existent for long-term memory (Kaplan et al., 1998).

Yet an alternative conceptualization (Jensen et al., 1997, 2006) conceives the features of ADHD as evolutionary advantageous traits derived from behavioral settings in ancestral or tribal hunter-gatherer societies where hypervigilance and hyperactivity, divided attention, and attentional scanning of the surrounding environment represent important parts of readiness to flight/fight response, which would augment the likelihood of survival. However, the latter is a hypothesis that still needs empirical confirmation.

One does not need champion evolutionary psychiatry, some other research, for example, has converged on the overlap between children who appear to be highly creative and children diagnosed with ADHD. Recent tests have shown that people with high levels of creativity often score low on measures of latent inhibition (Carson et al., 2003). It has been argued that it is because of lack of such type of inhibition that these individuals are able to problem-solve so creatively. It seems that these individuals have a genuinely different way to negotiate distinct competing sources of information and how it is then used for higher cognitive activities such as thinking: environmental cues seem to overwhelm the influence of internal elaboration, and produce vastly different responses than in less creative people. Specifically, Abraham and colleagues (2006) found that children with ADHD could overcome constraints of recently shown examples and create unique imagery. However, when asked to imagine an object from three different shapes, they failed to produce a practical one. That ability to think freely without the influence of rules or constraints is a fundamental characteristic of problem solving which has well supported neurocognitive basis (Shallice and Cooper, 2011), furthermore, several real-life case studies confirm it is associated with excellence in business, science, art and innovation (West, 2003). Although the evidence suggests promising possibilities, much empirical work is needed to flesh out the adaptionist perspective on creative reasoning and problem solving in individuals with ADHD.

The second example, dyslexia, is characterized as a reading learning disability. Generally, many children with dyslexia show language acquisition delays; they have trouble retrieving words from memory, mastering grammar, planning and organization. Moreover, they frequently have low processing speed, time awareness, focus and error detection (Pugh and McCardle, 2009). One of the most influential accounts for the symptoms is the phonological core account, according to which the difficulties with reading and writing stem from impairment in segmenting and discriminating patterns of phonemes (Stanovich and Siegel, 1994). Although such account considers individual differences, it seems that would not easily accommodate some of the other deficits found with dyslexia, such as poor finger coordination and eye movement while reading (Wolff et al., 1990; Bucci et al., 2012) and a very recently reported deficit in procedural learning which would make imitating and learning routines exceedingly difficult for children with dyslexia (Nicolson and Fawcett, 2000).

Geschwind (1982, 1984) and Geschwind and Galaburda (1987) were the first to propose an adaptionist view of dyslexia—and by virtue of the frequent comorbidity between the two, indirectly of ADHD as well. They proposed that genetics and in utero hormonal activity modified neurodevelopment and hemispheric specialization such that a person could be born with a brain wired to be at risk for dyslexia but at the same time with superior nonverbal abilities. They and others have proposed that the setting of the left hemisphere language areas to be prone to language-based impairments could in fact affect the growth of portions of the right hemisphere such that there might be an overrepresentation of nonverbal giftedness in samples of dyslexic individuals. Thus, on this view, gene-brain-environment interaction yields neural strengths and weaknesses, which, depending on the behavioral settings, can translate into socially defined talents and disabilities. Indeed, there is growing evidence that dyslexic individuals do seem to have selective, but very subtle and specific advantages for the span of visuo-spatial attention (Geiger and Lettvin, 1987), speed in performing global evaluation of complex visual stimuli (von Károlyi, 2001; Winner et al., 2001; von Karolyi et al., 2003), and visual comparisons (Schneps et al., 2007).

Early studies of dyslexia supporting Geshwind and Galaburda's thesis suggested that deficits in reading originate from an alternate neural circuit, which relies heavily on the right hemisphere (e.g., Yeni-Komshian et al., 1975). This hypothesis has since been modified and redefined as an interhemispheric connectivity issue affecting primarily the angular gyrus, which is hypothesized to be an important circuit linking visual association areas with language centers, especially, Wernicke's area (Horwitz et al., 1998). The lack of “short” interconnections in the angular gyrus may be explained by the broad spacing found in the minicolumns of dyslexic patients' brains by Casanova and colleagues (2002a). A slight change in the functional minicolumn structure would have widespread effects across the brain, impacting connectivity between areas of the brain, and, in doing so, information-processing functions (Casanova, 2010; Casanova et al., 2010). The alternative circuit in dyslexic patients may be the result of a distinct shift in processing, which appears to be most detrimental during phonological decoding (Simos et al., 2000). Other structural differences found in brains of individuals with dyslexia could be explained by a general change of minicolumn morphology. The brains of individuals with dyslexia show decreased gyrification, and atypical lateralizatization (Rumsey et al., 1997), as well as a wider gyral window (Casanova et al., 2010). Structural processes such as lateralization and gyrification may result from an addition of minicolomns within the isocortex (Casanova and Tillquist, 2008), and a wider gyral window allows for longer cortico-cortical fibers and spaced out minicolumns (Casanova et al., 2010). Only future research efforts will tell whether minicolumnar structure and organization are indeed linked to cognitive functions and preferences (Casanova, 2010), thereby, addressing some of the same limitations we have discussed in section “Issues about deficit attribution, cross-cultural neuropsychology and the integration of cognitive and brain activation levels of analysis.”

Can the adaptionist explanatory framework be extended to low-SES children? Consideration of such a possibility is scant but does exist in the literature, with supporting behavioral evidence (e.g., Buckner et al., 2009, 2003). Some neuroscientific support comes from aforementioned ERP studies on executive and selective attention in children living in poverty-stricken and inner city neighbourhoods. For instance, there is consistent accumulating evidence that Low-SES children attend both to relevant and irrelevant information employing in the process more intense and longer effortful control than high-SES counterparts (D'Angiulli et al., 2008b; Stevens et al., 2009). We have argued that in the behavioral settings in which these children live (chaotic, noisy, and threatening) indiscriminate attention and monitoring of environmental cues (“ear-to-the-ground”) may actually be adaptive for detecting dangerous or unwanted situations, to trigger in a top-down fashion appropriate fight/flight responses (i.e., D'Angiulli et al., 2008a,b). However, similarly to the evolutionary advantage of ADHD traits, the latter hypothesis also needs further empirical confirmation.

### Deficit attribution, disability and some ethical caveats of interventions

The ability of our brains to adapt to a quickly and constantly changing environment is one of the reasons why humans have become a complex species with a high range of adaptability (Bednarik, 2011). However, it is difficult to demonstrate the adaptive potential of many neural processes other than those involved in literacy and numeracy because until very recently, the emphasis has concentrated mostly on inter-group differences in those subjects. We should ask whether the difficulties to filter information normatively as shown by some low-SES children, for example, could also coexist with high imagination competency or higher spatial situational awareness and physical agility in their own environments not picked up by mainstream research methods (e.g., Nunes et al., 1993). The fact that laboratory tests may fail to mimic some potentially key ecological dimensions of real-life environments is just one confound that is not properly addressed and that could be improved with approaches combining longitudinal behavioral and neural activation measures in behavioral settings.

We do not condemn intervention efforts, but rather suggest a critical take on them, to integrate adequately and genuinely the contribution of many scientific approaches that responsibly are trying to improve ways to study childhood poverty, as per DCN community. For instance, instead of seeking to only change the child, the education system could benefit by adapting to accommodate for different neurocognitive preferences, such as DCN studies on individual variability show. For example, a study by Lawrence et al. (2002) on ADHD children in a natural environment showed that the deficit in working memory does not appear while the children were playing video games. They suggested that continuous feedback and reinforcement in the form of visual and auditory cues, as well as the opportunity to self-pace, alleviated the strain on working memory enough for the children to perform well. On the other hand, dyslexic children have an easier time with explicit learning and having the explicit rules broken down into manageable steps, instead of imitating a full routine. This kind of information would be helpful for teachers with not only dyslexic and hyperactive children, but also with all children who have trouble with working memory or procedural learning, including some low-SES children. This kind of approach can help lessen social pressures on low-SES children in contexts such as those inducing the pygmalion effect (Rosenthal and Jacobson, 1968).

The effect of a teacher's bias on the performance of children in the classroom has been known since many years ago (e.g., de Boer et al., 2010) and although the original pygmalion effect needs substantial revisions, the most recent reviews confirm that stigmatized groups such as African-Americans and children from low-SES backgrounds still continue to be sensitive to the expectations of their teachers, and do poorly when there is an assumption of failure (McLoyd, 1998; Jussim and Harber, 2005) spending extraordinarily more energy negotiating for their image in society than their middle- or high-class counterparts (Stephens, 2010) with increased odds of learned hopelessness or helplessness (Ursin and Eriksen, 2010).

Thus, it is important to consider and reflect about the responsibilities of the scientific community to prevent harmful myths, prejudices, misconceptions, and how some research may be, even if unwillingly, contributing to them. If some of the assumptions and attributions we consider here may not only be seen in the case of low-SES children, being connected with the conceptualization of developmental disabilities such as ADHD and dyslexia, we could observe again a pattern where “difference” seems interpreted as “deficit.” Nevertheless, based on what we learn in the comparison with these disabilities, the conditions associated with low-SES could at some level be defended as having an adaptive role even considering a deprived environment—which definitively implies an unethical circumstance.

If interventions can be created which have an impact on underlying neural processes, the crucial questions still remain: do we want to change the atypical neural preferences to normative ones? And in such a case, as it may apply to low-SES children, what are the foundations of our intentions as scientists? Are we being careful in not hastily putting the horse of intervention before the cart of understanding? These and other poignant questions regarding interpretation and translation of research results, as well as their far reaching implications, overlap remarkably with the most recent debate regarding ethical, legal and social issues in neuroethics (Illes et al., 2006). However, while the debate over the clinical use of neuroimages is right now very much alive, a parallel debate on the neuroethics of intervention is overdue in the DCN of SES.

### Limitations of the adaptionist perspective

The adaptionist perspective is not immune to problems. Thus, to rebalance our critical review, we need to consider a few of its shortcomings here. One major issue is that very few research efforts have consolidated the adaptionist evidence-base, and most of the hypotheses need much more empirical testing and corroboration (as we did note in previous sections). The adaptionist perspective is based on an underlying assumption of complexity that preserves a general determinism (for example, probabilistic as in dynamic systems). However, cannot be mapped or decomposed easily into strict causality relationships (Bunge, 1979). Therefore, it does not offer very transparent explanations that give compelling accounts for indirect effects associated with low-SES environments. For example, it is not clear how certain variables associated to low SES such as inadequate nutrition can be incorporated in adaptionist models. Neurobiological and neurophysiological studies have shown that a poor diet during pregnancy and/or during the infants' first months of life can induce anatomic and functional development problems in the brain (e.g., Morgane et al., 1993, 2002; Georgieff, 2007). In some cases, low-SES participants may be facing real brain deficits that later might have irreversible consequences on their performance on cognitive tasks (e.g., de Souza et al., 2011; Waber et al., 2011; Galler et al., 2012). The same goes for the neurotoxic effects of theratogens and pollutants (see section “Issues about the definition of poverty”). Which aspects could be considered adaptive in these circumstances? If adaptionism is not related to the ecology of the living conditions (the behavioral settings) then the explanatory power of that approach is quite limited.

Although there is no need to link adaptions to evolutionary advantages, which by themselves have problems of falsifiability and possible ideological confounds (e.g., the implicit assumptions of positivistic progress, see Searle, 1995), it is difficult to empirically validate what is or not adaptive in a given situation, and decisions on what is or not adaptive may ultimately appear to be nothing but arbitrary.

Related to the previous criticisms, one implication is that the adaptionist perspective can be easily misinterpreted as a form of extreme relativism minimizing real challenges that certain groups are likely to encounter. This in turn may actually be harmful to children who do have actual deficits and do actually need support.

In sum, adaptionism can be as speculative as the deficit assumption. If misinterpreted or misused, it may be perceived as or actually become a roadblock to advances in the field of intervention. However, some aspects of the definition of adaptations can be improved and falsifiable, we explore further these features in the next section to propose a framework in which they can constructively enhance, not stonewall, the approach to interventions.

## Some possible alternatives and future directions

### Experimental interventions and real deficits

If adaptionism is properly examined in ecological settings, the idea of deficit can be redefined as a variable set of environmental conditions experienced by low-SES participants which becomes a detrimental factor in preventing optimal brain development, possibly resulting in irreversible neurocognitive impairment. This conceptualization does not underestimate the possibility of “real” deficit; and permits to incorporate experimental interventions as a way to measure deficit in a non-normative fashion. That is, experimental interventions could be the main tool to determine the extent of reversibility, and conversely, plasticity, since with the appropriate intervention a low-SES or poor child's performance could catch up with that of the higher SES counterparts. In this way, it would possible to identify real and essential deficits as a miss or break down of adaptation. Accordingly, possible neurocognitive deficits could be identified without sidestepping ethical issues (since intervention may be offered to children from all SES background as a preemptive form of support), and without relying on value-laden normativity criteria. Conversely, such approach would also permit to properly evaluate the extent of deficit even in some instances of extreme early or chronic deprivation, for example some forms of malnutrition whose effects are at least partially reversible with early intervention (e.g., Martorell et al., 1994).

### Toward an ecological developmental cognitive neuroscience framework

Approaching the implications and issues that deficit attribution and normativity have in the context of an interdisciplinary DCN research agenda, focused on child development, biological and social determinants, implies starting from a wide conceptualization. Such framework should make provisions for multiple levels of analysis, explanations in terms of interactive complex mechanisms situated in turn within a systemic, comprehensive, and coherent approach incorporating ecological perspectives (e.g., Barker, 1968; Bronfenbrenner and Ceci, 1994). Thus, we propose that an ecological developmental cognitive neuroscience (eDCN) framework would be necessary to promote a visualization of child development, developmental processes and social determinants thereof as complex phenomena. The construction of a common language dealing with child development and determinants (i.e., biological, social, and cultural) in ecological terms would be a first, necessary step toward the construction of academic networks apt at informing about both the design, and implementation of comprehensive, coherent experimental interventions.

Consistent with eDCN, ecological and transactional considerations on child development and determinants should contribute to build a research agenda with at least the following updated issues:

- *Identifying different problems and risk factors levels for both basic and applied research*. For example, considering ecological theories, the complex set of problems and risk factors characterizing the biological, psychological and cultural determinants of low-SES child development would offer researchers a basis for organizing research agendas, with focus on development as a complex phenomenon, being integrated into different conceptual, methodological frameworks, and being applicable within different, sociocultural contexts.
- *Reconceptualizing measurement of child poverty*. Conceptual and operational definitions of poverty are unlikely to capture either specific information on the deprivation children are subjected to, or to associate deprivation with different developmental phases and dimensions. Specifically the level and type of deprivation, as well as brain developmental stage at the time of deprivation, may modulate the impacts. As mentioned, several studies have advised that children exposed to poverty must be taken into account as independent analysis units as they are exposed to poverty's effects in a different way than adults or other children (Gordon et al., 2003; Roosa et al., 2005; Minujin et al., 2006). Although these studies improve the comprehension of how poverty affects child development, they still do not take into account the interrelationship and interdependence between phases, contexts, and dimensions of development. At present, many researchers agree that the multifactorial nature of poverty means that multidimensional measurement methods have to be adopted and adapted to child poverty. Such a demand for multidimensional definitions and measurements imposes an obligation to carefully select the measurement methods: questions, hypotheses, and study objectives aimed at analyzing child development processes in both research to identify, describe, and research into intervention strategies.
- *Guiding the design of interventions in terms of different systems and dimensions involved in child development components and processes*—i.e., building an ecology of interventions through data-driven theory. In particular, the current research agenda in the DCN area suggests there is a need to analyze the emergence of different self-regulatory, cognitive, and emotional processes, their role in school learning process, their modulation in function of parents' mental health and home stimulation as well as their potential optimization through home, school, and community (Lipina and Colombo, 2009). However, we propose that this should be done systematically within a common research framework to all researchers and guided by a process of knowledge building through the formulation and falsification of data-driven theories.
- *Focus on promoting support for both basic and applied research on child development as the main focus.* Although financial institutions and organizations—especially those in developing countries—usually consider child development issues to be a high-priority area, there exists a lack of visualization of the complexity of such mechanisms and determinants. In addition, several types of academic inertias act in favor of some approaches at the expense of others (e.g., either Constructivism vs. Behaviorism in education-based research or environmental vs. genetic determination of cognitive competences throughout development). The latter, reduces possibilities for genuine disciplinary integrations as well as researchers' formation in new areas such as neuroscience and education. In this context, supporting efforts aimed at promoting collaborations focused on different levels of analysis would be important, such as: (1) modulation of parenting dealing with the development of self-regulatory competences; (2) analysis of the associations between teaching styles, and the development of executive control competences; (3) identification of cultural constraints for nutritional supplementation interventions; (4) integration of cognitive, emotional, and social competences stimulation when designing school curricula; (5) inclusion of art in community interventions as a tool for social and health transformations, such as for example *El Sistema* in Venezuela; or The Children Harlem Zone in New York (6) knowledge mobilization of intervention approaches across the globe.
- *Building capacity aimed at progressively eliminating myths, prejudices, as well as conceptual dogmatisms*. As mentioned, the lack of visibility and vision for childhood is a frequent problem in many countries worldwide that involves parents, teachers, politicians, and policy-makers. Nevertheless, it is possible to find an even more serious situation in academia. This implies an urgent need to create new instances of formation at the local and global scales.
- *Influencing public opinion, through the media, to promote collaboration between researchers and journalists, based on the consideration of child development as a complex, systemic phenomenon.* Part of the researchers' social responsibility lays in disseminating knowledge—hence avoiding myths to be created with regard to child development. Thus, setting up common, ethical norms so that public opinion could be informed through the media would be not only feasible but also desirable. Furthermore, training efforts could be generated such as interdisciplinary workshops, and debates—and, even, contents useful to university degree courses for Social Communication and Journalism programs as well (see Illes et al., 2010). Such efforts should also be integrated within multiple modules of intervention designs, from an ecological, transactional perspective, for example, as an integral component of activities guided by the proposed eDCN framework.

## Conclusions

By no means do we argue that cognitive and behavioral performance differences between low- and high-SES children are mere epiphenomena of contrived laboratory experiments or that such differences play no part in determining children's economic and social outcomes in adulthood. Such differences have been well documented across different cognitive domains, using a variety of experimental designs and cognitive tasks. Thus, these empirical findings deserve utmost attention of developmental cognitive neuroscientists, as they are suggestive of differences in structural and functional organization of the brain as a function of the SES context in which it develops. In addition, the hypothesis that observed differences are largely attributable to cognitive deficits in low-SES children should be considered with great caution, since it could ignore the fact that different SES contexts present unique environmental challenges to which children must learn to adapt. Therefore, it seems more likely that at least some performance differences between low-and high-SES children reflect differences in structural and functional organization of the brain as a result of context-specific organism-environment interactions which potentially confer different forms of adaptions to their own environmental settings.

Taking this conceptualization seriously, it is possible to recognize that positive adaptions to one specific environment may not be generalizable to other environments, and therefore may not be congruent with them in terms of certain of their cognitive, behavioral, and socio-emotional requirements. By extension, using developmental norms of one context to assess cognitive, behavioral and socio-emotional performance of children from another context may reveal clear performance differences plausible to be interpreted as deficits. Such misinterpretation, however, is relative to diverse contexts taken as normative and therefore biased and wrong because it does not explain cognition and behavior in ecologically valid terms.

There is a variety of deprived circumstances characterized by specific ecological risk factors present in moderate and severe low-SES settings (e.g., Gordon et al., 2003) which predispose children to particular types of developmental insults, with potentially irreversible neurological consequences. A number of such risk factors have already been documented, including malnutrition, exposure to environmental toxins and severe physical and psychological abuse (Bradley and Corwyn, 2002; Lipina and Colombo, 2009). However, research in this area has exclusively focused on low-SES environments, despite the recent evidence that high-SES contexts may too predispose children to certain negative cognitive, behavioral and socio-emotional impacts, perhaps different in kind, but no less developmentally important (Luthar and Ansary, 2005; Luthar, 2006; Ansary and Luthar, 2009). Therefore, both negative and positive ecological factors should be considered at all levels of the SES spectrum.

Furthermore, it is not the purpose of the paper to discourage any type of effort to develop and implement intervention programs aimed at optimizing both cognitive and socio-emotional development of children from low-SES backgrounds. Any improvements in academic performance, reduction in grade repeating and/or school failure are considerable steps forward in influencing both social and economic outcomes of children who grow up in circumstances which do not readily afford them sufficiently appropriate developmental opportunities. In addition, it is clear that such programs can be rich sources of empirical data concerning the mediating effects of cognitive and behavioral training on the structure and functioning of the brain, constituting from the eDCN perspective, valuable information on experience-dependent neuroplasticity.

Finally, we are optimistic about the role that cognitive neuroscience can play in the solution of the problem of the relationship between brain development and SES disparities. Ultimately, such a solution must include an explanation of how different social and economic conditions in which children grow up differentially influence the growth and development of neural mechanisms, and how such differences translate into adult neurocognitive outcomes. At a more practical level, cognitive neuroscience in collaboration with other disciplines can greatly contribute to the design of practical methods to deal with various developmental issues associated with different SES contexts. Both, the field's current and future technological and methodological advances are promising in addressing these questions. But probably those alone will not be enough. Substantial advances need to occur most and foremost at the level of theoretical conceptualization and underlying epistemology. The analysis and proposals reviewed here may contribute to a debate steering the field a step forward in the right direction.

### Conflict of interest statement

The authors declare that the research was conducted in the absence of any commercial or financial relationships that could be construed as a potential conflict of interest.
